# Adjuvant Therapeutic Modalities in Primary Small Cell Carcinoma of Esophagus Patients: A Retrospective Cohort Study of Multicenter Clinical Outcomes

**DOI:** 10.1097/MD.0000000000003507

**Published:** 2016-04-29

**Authors:** Bingwen Zou, Tao Li, Qiang Zhou, Daiyuan Ma, Yongshun Chen, Meijuan Huang, Feng Peng, Yong Xu, Jiang Zhu, Zhenyu Ding, Lin Zhou, Jin Wang, Li Ren, Min Yu, Youling Gong, Yanying Li, Longqi Chen, You Lu

**Affiliations:** From the Department of Thoracic Oncology (BZ, MH, FP, YX, JZ, ZD, LZ, JW, LR, MY, YG, Y Li, Y Lu), Cancer Center, West China Hospital/Medical School, Sichuan University; Department of Radiochemotherapy Oncology (TL), Sichuan Provincial Cancer Hospital, Chengdu; Department of Oncology (QZ), Suining Center Hospital, Suining; Department of Radiotherapy Oncology (DM), Affiliated Hospital of North Sichuan Medical College, Nanchong; Department of Radiotherapy Oncology (YC), Henan Provincial Cancer Hospital, Zhengzhou; Department of Thoracic Surgery (LC), West China Hospital/Medical School, Sichuan University, Chengdu, China.

## Abstract

To evaluate the treatment pattern and survival of patients receiving radical resection for primary small cell carcinoma of the esophagus (PSCCE).

This retrospective study included 150 patients who received radical resection of PSCCE. Data were retrieved from 4 centers in Western China. Thirty-nine of 150 patients received postoperative chemo-radiotherapy, 62 received postoperative chemotherapy, and 49 received radical resection only. The median radiation dosage was 50 Gy. The chemotherapeutic regimen was platinum-based and lasted for 2 to 6 cycles (median, 3).

Median disease-free survival (mDFS) and overall survival (mOS) were 12.0 and 18.3 months, respectively. Subgroup analysis revealed that postoperative therapy did not improve survival in limited stage I (LSI) disease, whereas postoperative chemotherapy improved survival in limited stage II (LSII) disease. Relative to chemotherapy alone, chemoradiotherapy did not improve survival in patients with completely resected LSII disease. A multivariate analysis indicated an association of no postoperative chemotherapy with shorter DFS (*P* = 0.050) and OS (*P* = 0.010). Higher lymph node stage and length of disease longer than 3 cm were poor prognostic factors for both DFS and OS.

Adjuvant chemotherapy improves survival in PSCCE patients with completely resected LSII disease. Adjuvant treatment with postoperative chemotherapy alone or postoperative chemo-radiotherapy does not increase survival in completely resected LSI disease.

## INTRODUCTION

Primary small cell carcinoma of the esophagus (PSCCE) is a rare and aggressive malignant tumor characterized by early metastases and a rapidly fatal course.^1^ Due to the lower incidence between 0.5% and 2.4% of primary oesophageal malignancies of this disease,^1–8^ large-scale analysis has rarely been reported since the first case was reported by McKeown in 1952. To our knowledge, the largest retrospective analysis of this disease included 151 patients who received varying treatments, and only 94 patients received surgery.^9^ Although several reports concluded that surgical resection of the localized low-volume form of the disease can result in long-term disease-free survival (DFS),^10,11^ most patients treated with surgery alone had rapid systemic recurrence.^12,13^ A literature review of 199 PSCCE patients demonstrated that adding systemic therapy to local treatment prolonged the median survival for 15 months for the limited stage disease;^8^ however, its role in postoperative localized disease is unclear, and the data for chemo-radiotherapy is limited thus far.^12^ There were no standard treatments established for PSCCE until now. Further investigation is warranted for new therapeutic modalities for PSCCE patients with dissection of the disease. The roles of postoperative chemotherapy and radiotherapy must be assessed in patients with resection of PSCCE.

Currently, it is necessary to evaluate the role of postoperative chemotherapy and radiotherapy and then to determine whether clinicopathological variables, such as lymph node (LN) stage, are predictors. In this retrospective multicenter study, 150 patients who underwent complete resection of PSCCE from 4 clinical centers in Western China were reviewed and analyzed. The present study assessed the benefit of postoperative chemotherapy and chemo-radiotherapy in primary endpoint OS as well as the secondary outcome DFS in local stage II disease.

## METHODS

### Patients

This retrospective, multicenter analysis included 150 patients with pathologically confirmed PSCCE from 4 cancer centers in Western China between May 1998 and September 2014. The histologic criteria applied for the diagnosis of small cell carcinoma were proposed by the World Health Organization.^14–16^ PSCCE was defined as disease originating from the esophagus with a normal computed tomography (CT) scan of the pulmonary system. Minimal staging procedures for all patients included history and physical examination, a bone scan, an MRI of the brain, a barium swallow, and a contrast CT of the chest and abdomen. Patients were assigned to a T and M stage in accordance with the American Joint Committee on Cancer TNM Classification of Carcinoma of the esophagus and Oesophagogastric Junction (7th ed., 2010). Cases without regional lymph node involvement were defined as N_0_, those with 1 to 3 regional lymph nodes involved were defined as N_1_, and those with more than 3 positive regional nodes were N_2_. The limited stage was defined as a tumor grown in the organ of origin and the loco-regional lymph nodes, which was easily encompassed within 1 radiation therapy treatment portal.^17^ Individuals with T_1–2_N_0_M_0_ were defined as limited stage I (LSI), whereas persons with T_3–4_N_0_M_0_ or T_1–4_N_1–2_M_0_ were defined as limited stage II (LSII).

The inclusion criteria were as follows: patients with R_0_ dissection and system mediastinal lymphadenectomy and patients between 18 and 75-year old and with Kamofsky performance status score of more than 70. Patients were excluded from the analysis if they had other malignancies and any serious concurrent diseases, such as serious chronic obstructive pulmonary disease, severe diabetes, or uncontrolled hypertension, or any residual tumors. The study protocol was approved by the local Institutional Review Board at the authors’ affiliated institution, and patient consent was not required because of the retrospective nature of the study.

### Patient Evaluation

We reviewed the updated information of 150 cases for overall survival, date of disease relapse, date of death or last follow-up, details of treatment, number of dissected lymph-nodes, number of positive nodes, sex, age and tumor stage.

### Treatment

Because of the lack of a recommended therapeutic schedule, the choice of treatment was mainly based on physician decisions, with some consideration of the economic situation of the patients. Sixty-two of 150 patients were referred to cancer centers for postoperative chemotherapy, whereas 39 patients received postoperative chemo-radiotherapy.

### Surgery

In our analysis, all thoracic surgeons performed oesophageal cancer resection through a left thoracotomy and 2-field lymph node dissection and had at least 10 years of surgical experience. Systematic and complete dissection of the mediastinal lymph nodes was performed in all cases for curative intent.

### Postoperative Chemotherapy and Radiotherapy

One hundred one patients received platinum-based chemotherapy, given with radiotherapy or alone. The regiment choice of chemotherapy was performed according to the doctor's decision, with reference to the treatment of oesophageal cancer and small cell lung cancer, including etoposide, paclitaxel, and irinotecan.

Radiation was delivered with 6 MV-X rays at 2 Gy per fraction, 5 days per week, with a total dose ranging from 44 to 50 Gy. The clinical target volume (CTV) for treatment encompassed the mediastinum. The planning target volume was determined as the CTV plus 1-cm margins. The radiation region extended from the oesophagogastric junction to the supraclavicular fossa, including the mediastinum, except when the radiation of the supraclavicular fossa was not required in patients whose cancers originated in the lower third of the esophagus. The exact placement of the field borders varied from case to case depending on the postoperative shift of the mediastinal structures and the length of the lesion. Early radiotherapy (ER) was defined as a regimen in which patients received postoperative radiotherapy within 60 days since the day they received surgery. Late radiotherapy (LR) was defined as a regimen in which patients received postoperative radiotherapy over 60 days since their operation.

### Follow-Up

Patients were followed up once every 3 months for the first 2 years, once every 6 months for the third year, and once yearly from the fourth year.

### Definitions and Statistical Analysis

Long-term outcome was determined from hospital records and follow-up information. Disease-free survival was measured from the date of surgery to the time of the first local or distant progress or the time of death from any cause. Overall survival was calculated from the date of surgery to death or the last follow-up visit. Local recurrence-free was defined as tumor relapse in the organ of origin and locoregional lymph nodes as visualized by CT scan or positive gastroscope. Recurrence beyond those sites was deemed distant progress.

Descriptive analysis was performed using univariate statistics to report the median and 95% confidence intervals for the continuous variables and frequency distribution for the categorical variables. Because of the potential bias of treatment, the patients were divided into 2 groups for analysis according to the stage. A *χ*^2^ test or Fisher exact test was performed for patient baseline characteristics. DFS and OS rates were calculated using the Kaplan–Meier method,^18,19^ whereas the cases without end events were censored. The survival curves were compared using the log-rank test in the univariate analysis, and a *P* value less than 0.2 was used for significance, so that factors were not missed in the multivariate analysis.^18^ Multivariate analysis was performed using Cox regression.^20^ S-CT was used as the baseline for therapeutic modalities and the multivariate analyses were stratified by LS stage. A *P* value less than 0.05 was considered significant. Univariate and multivariate analyses were conducted to compare the survival between groups using Cox proportional hazards with SPSS 13.0 (SPSS Inc, Chicago, IL).

## RESULTS

### Treatment Characteristics

One hundred one patients received chemotherapy with a median of 3 cycles (range, 2–6). Sixty-nine patients received etoposide (60 mg/m^2^ intravenously, on days 1–3) and cisplatin (25 mg/m^2^ intravenously, on days 1–3) with a median of 4 cycles (range, 2–6); 23 patients received paclitaxel (135 mg/m^2^ intravenously, on day 1) and cisplatin (25 mg/m^2^ intravenously, on days 1–3) with a median of 4 cycles (range, 2–5); and 6 patients received cisplatin (25 mg/m^2^ intravenously, on days 1–3), leucovorin (200 mg/m^2^ intravenously, on days 1–5), and fluorouracil (750 mg/m^2^ intravenously, on days 1–5) with a median of 3 cycles (range, 1–4). Three patients received irinotecan (60 mg/m^2^ intravenously, on days 1, 8, and 15) and cisplatin (25 mg/m^2^ intravenously, on days 1–3) with 2 to 3 cycles.

Of these, 39 patients received radiotherapy, and 28 patients received three-dimensional computerized dosimetry planning and radiotherapy.

### Patient Demographic and Baseline Characteristics

The patient ages at diagnosis ranged from 39 to 75 years, with a median age of 59. The clinical characteristics at presentation are outlined in Table 1 and were well balanced.

**TABLE 1:**
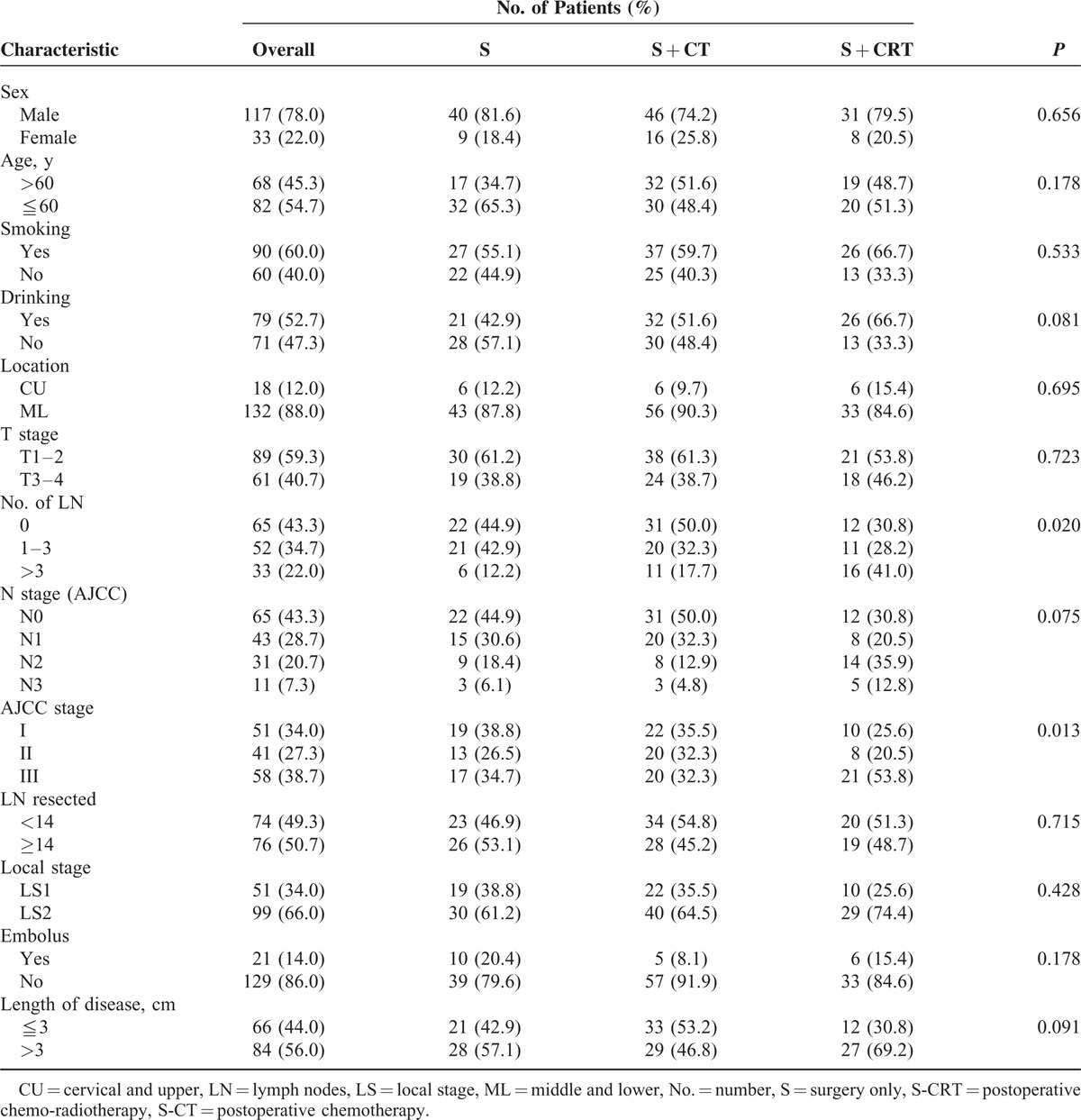
Patient's Characteristics

### OS and DFS

**FIGURE 1:**
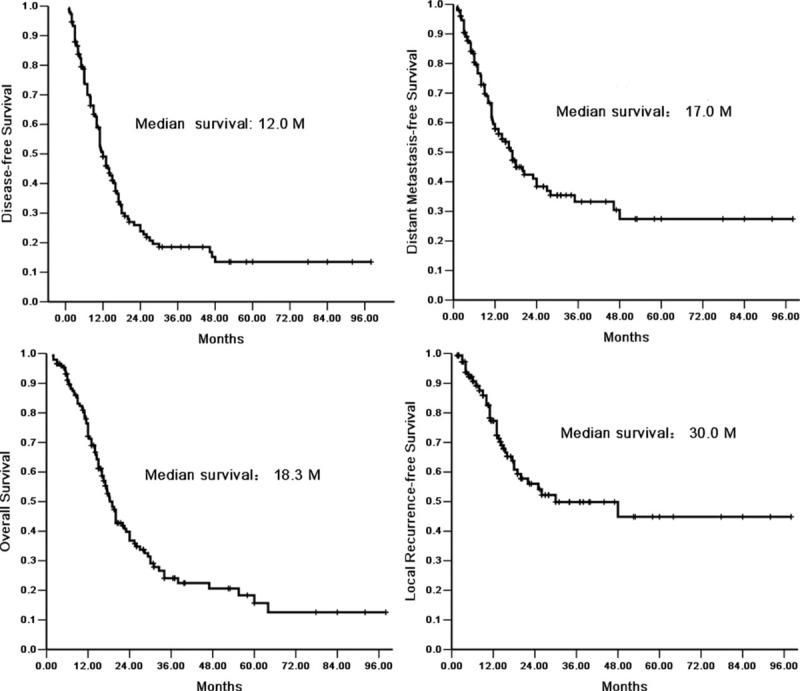
Survival for all patients.

The median DFS of all patients was 12.0 (95% CI: 9.8–14.2), and there were no significant differences in the DFS among the S, S-CT, and S-CRT groups (*P* = 0.264). The median OS was 18.3 (95% CI: 16.2–20.4) months, whereas the OS did not differ significantly among the 3 groups (*P* = 0.055) (Figure 1).

According to the NCCN guidelines (Version 2.2014) for limited stage small cell lung cancer, the recommended treatment for T_1–2_N_0_M_0_ stage disease is entirely different from other limited stage diseases. We stratified the patients to LS I and LS II. Further analysis was performed on the LS I disease patients (Table 2). The OS, DFS, LRFS, and DMFS did not differ significantly among the 3 groups in LSI patients (Table 3), whereas some favorable findings were noted in LSII disease. For patients with LS I disease, there was no significant benefit in survival for patients who received postoperative chemotherapy or chemo-radiotherapy.

**TABLE 2:**
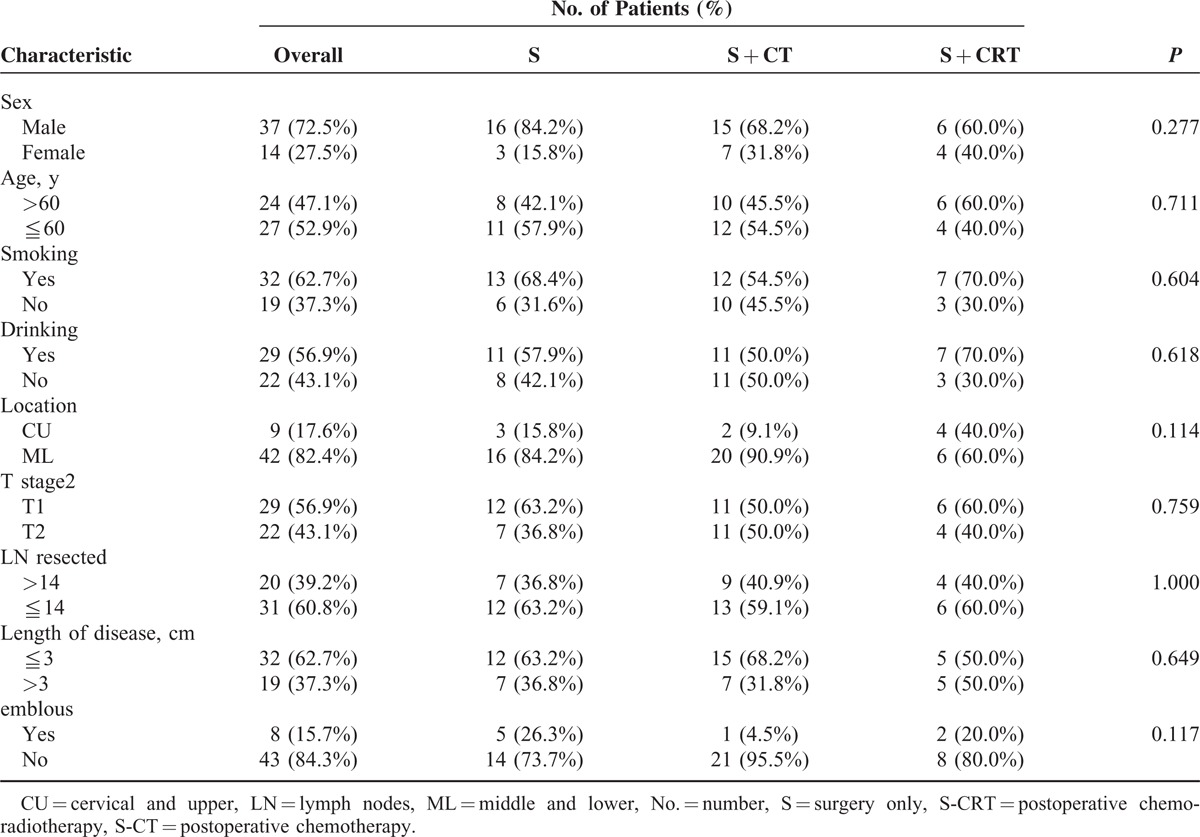
The Clinical Characteristics of the LSI Patients

**TABLE 3:**
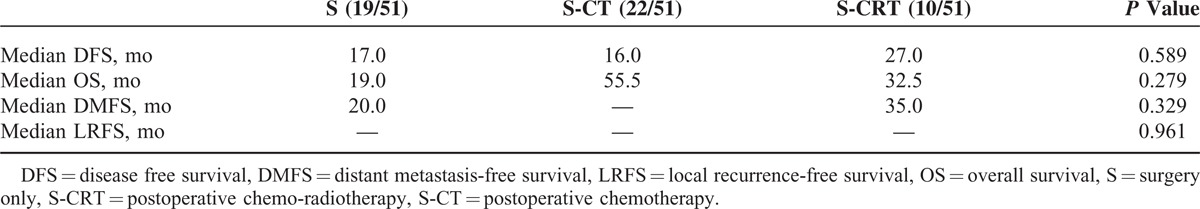
Univariate Analysis of the Prognostic Factors for Survival in Limited Stage I Patients

### Subgroup Analysis of Patients With Limited Stage II PSCCE

**FIGURE 2:**
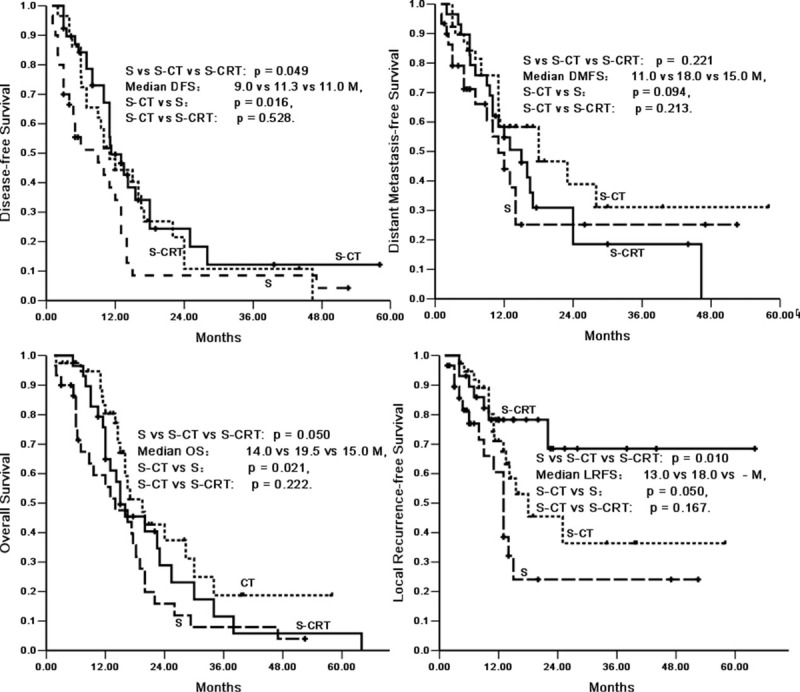
Survival stratified by postoperative chemo-radiotherapy, postoperative chemotherapy, and surgery only in limited stage II patients.

Subgroup analysis showed that in patients with limited stage II disease (Tables 4 and 5), there were apparent divergences in DFS (median DFS, S vs. S-CT: 9.0 vs. 11.3 months, *P* = 0.029), OS (median OS, S vs. S-CT: 14.0 vs. 19.5 months, *P* = 0.021), and local recurrence-free survival (median LRFS, S vs. S-CT: 13.0 vs. 18.0 months, *P* = 0.050) between the S and S-CT groups, whereas there was a tendency for distant metastasis-free survival in both groups (median DMFS, S vs. S-CT: 11.0 vs. 18.0 months, *P* = 0.094) (Figure 2).

**TABLE 4:**
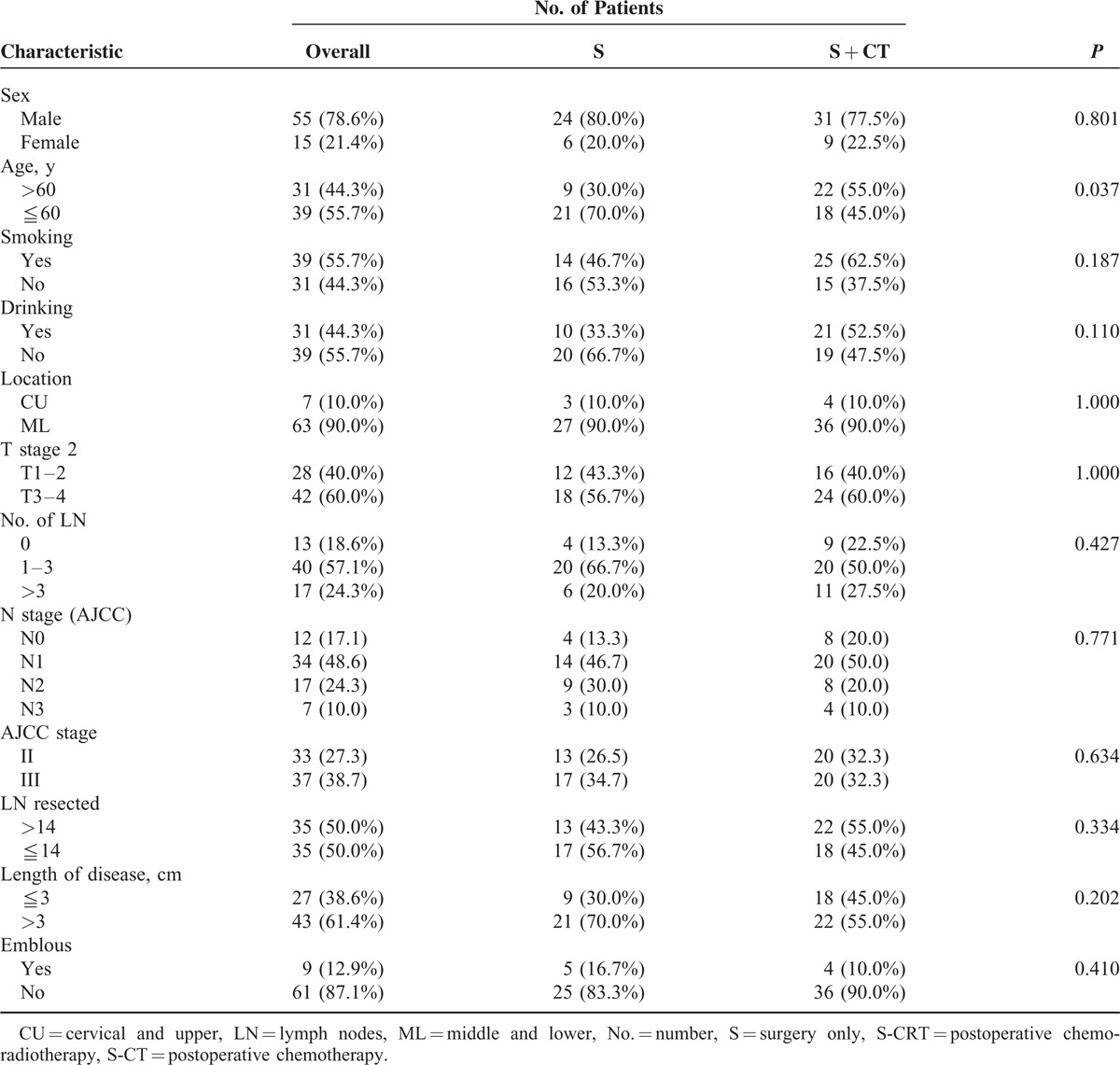
The Clinical Characteristics of the LSII Patients Received S and S + CT

**TABLE 5:**
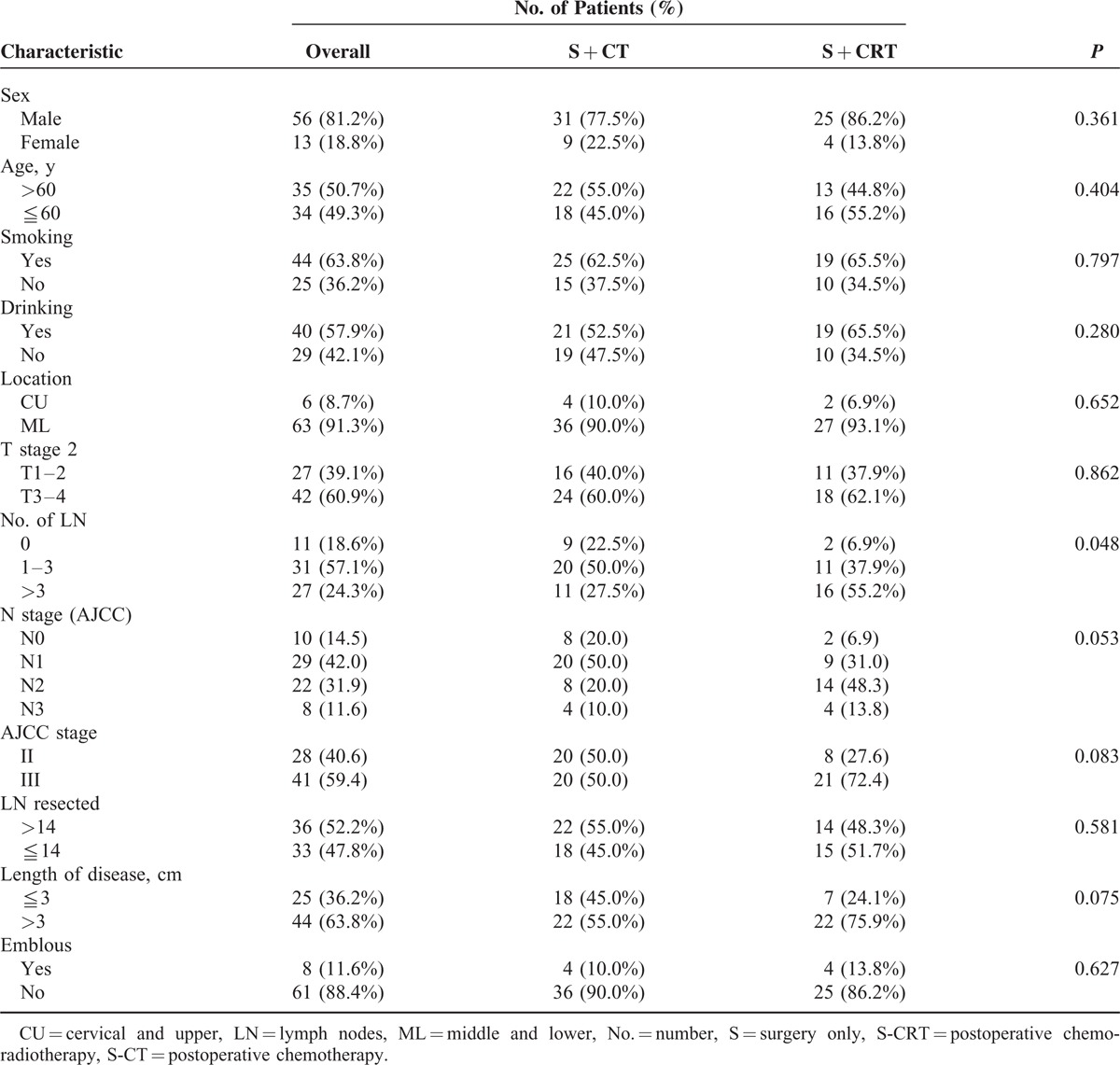
The Clinical Characteristics of the LSII Patients Received S + CT and S + CRT

By contrast, when radiotherapy was added to postoperative chemotherapy (S-CRT) in limited stage II disease patients, there were no significant divergences in overall survival and disease-free survival compared with postoperative chemotherapy (S-CT) (Figure 2). Local recurrence-free survival and distant metastasis-free survival were compared between the S-CT and S-CRT groups. The median LRFS was 18.0 months for the S-CT group compared with the S-CRT group (*P* = 0.167), whereas the median distant metastasis-free survival was 18.0 and 15.0 months, respectively (*P* = 0.213) (Figure 2). The overall recurrence rate in the S-CT was 65.0% (26/40), whereas in the S-CRT group it was 82.8% (24/29) (*P* = 0.103). Furthermore, there were no significant differences in the local recurrence rate (LRR) between the S-CT and S-CRT groups (40.0% vs. 24.1%, *P* = 0.168). However, the distant metastasis rates (DMRs) were 47.5% and 72.4% (*P* = 0.038), respectively.

An additional study was conducted for all 29 limited stage II disease patients who received postoperative radiotherapy. The DMRs between the ER group and the LR group were 85% (17/20) and 44.4% (4/9) (*P* = 0.067), respectively, which favored the late radiotherapy group. There was no significant difference in the rate of N_2_ stage disease between the ER and the LR groups (14/20 patients in the LR group and 4/9 patients in the ER group for N2 stage, *χ*^2^ = 1.722, *P* = 0.189).

### Prognostic Factors

The prognostic factors for all patients are described in Table 6. In the multivariate analysis compared with the S-CT, no postoperative chemotherapy (S) had an adverse predictive value for both OS and DFS, whereas the S-CRT did not influence survival. The risk of the death and disease progression increased by 94.0% and 61.3%, respectively, for the patients who did not have postoperative chemotherapy. Lymph node stage (N_0_ vs. N_1_ vs. N_2_) and the length of disease (≤3 cm vs. >3 cm) significantly influenced both OS and DFS when embolus (yes vs. no) was substantial to the overall survival.

**TABLE 6:**
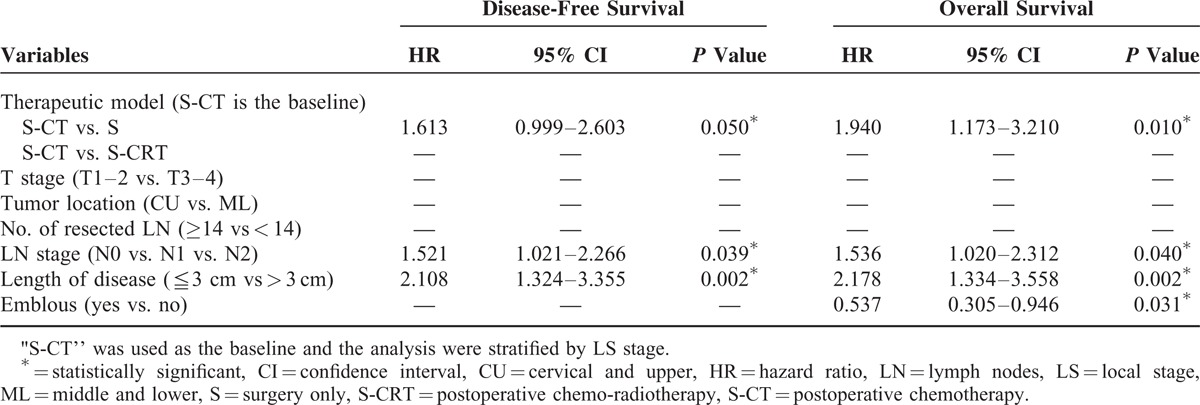
The Results of Multivariate Analysis in Different Prognostic Factors for All Patients

### Patterns of Failure

After a median follow-up of 17.3 months for live patients, 96 patients died. Ninety deaths (93.8%) were cancer-related or cachexia, 4 patients died of lung infection (4.2%), 1 patient died of haemorrhage of the upper digestive tract, and 1 patient died of postoperative complications of tongue cancer. To date, 50 patients relapsed, 80 patients developed distant metastatic disease, and the cause of failure was unknown for 3 patients. Thirty-seven patients had liver metastases, 24 had lung metastases, 10 had lymph node metastases, 8 had bone metastases, 9 had brain metastases, and 1 had skin metastasis. Multiple distant metastatic sites were observed in 7 of these 80 patients.

## DISCUSSION

Chemotherapy and radiotherapy are accepted as part of the treatment for patients with PSCCE, including those with R0 dissection disease,^1,8,11–13,21–23^ although the effect of adjuvant chemotherapy and radiotherapy remains unclear.^12^ To our knowledge, this is the first and largest multi-institution study to evaluate postoperative chemotherapy and radiotherapy in patients with completely resected PSCCE.

In our study, although some patients did not receive postoperative therapy mainly due to economics, the median DFS was 15.0 months for all cases, which is better than a previous result.^9^ This may be due to the inclusion of cases with R0 dissection and good Kamofsky performance status in our study. Postoperative chemotherapy and postoperative chemo-radiotherapy were not prognostic factors in the univariate analysis for limited stage I patients. Previous studies focused on chemotherapy or chemo-radiotherapy for improving survival, but few noted the category of LS patients who are favored by these treatment modes.^24–27^ In the present study, there was no significant benefit in survival for patients who received postoperative chemotherapy or chemo-radiotherapy for limited stage I patients compared with those with surgery only (Table 3). A similar effect of postoperative chemotherapy and chemo-radiotherapy was presented by others for limited stage small cell carcinoma of esophagus.^5,10,12^ Mitani et al.^5^ reported that 2 of 3 T_1_N_0_M_0_ primary small cell carcinoma patients who had radical dissection remained disease free for more than 7 years. In another report, 2 of 9 patients with limited stage disease who received surgery survived for longer than 5 years.^10^ A previous study of PSCCE emphasized that in certain circumstances, surgical resection may be associated with a favorable outcome, such as for patients with localized low volume disease, which was similar to the results of our study.^12^

When comparing the groups receiving and not receiving postoperative chemotherapy, notable improvement of OS and DFS was demonstrated in limited stage II patients who received postoperative chemotherapy. The survival advantage associated with S-CT in this analysis was consistent with that of previous reports.^8,12,27^ Chemotherapy gave better local recurrence-free survival and a trend of distant recurrence-free survival, which resulted in improved DFS and OS, when S-CT was compared to S only.^8,12^ The survival curves for patients receiving postoperative chemotherapy and for those receiving surgery only show an obvious divergence from the beginning of follow-up (Figure 2). This suggests that improvement in LRFS and DMFS is achieved by a therapeutic regimen of chemotherapy, leading to improved DFS, and ultimately, to an improvement in OS. A similar effect of S-CT was presented in a previous analysis for patients with PSCCE.^24,25^ Several authors indicated that surgery with chemotherapy should be considered as the curative choice because the better survival was reported in dissected patients, which was consistent with our results.^12,24^ In the present study, several prognostic factors were assessed for all patients, whereas S-CT was an independent prognostic factor for both OS and DFS in the patients receiving anticancer treatment. Patients without chemotherapy had significant adverse effects on both OS and DFS in all patients, in contrast to the patients who received chemotherapy. Those who received surgery only had 1.9 times the risk of death and had 1.6 times of the risk of disease progression. The survival advantage associated with S-CT in our analysis was consistent with several previous reports.^25,28,29^

When compared with the group who had S-CT alone, there were no survival advantages for both the OS and DFS among the limited stage II patients who received a combination of postoperative radiotherapy and chemotherapy. No survival benefit was associated with S-CRT in our analysis, contrary to several previous reports.^6,12^ According to previous research, S-CRT had significantly better LRFS,^9,13,21,22,24,25^ which resulted in significantly improved DFS and OS when S-CRT was compared with S-CT.^26,27^ However, there was no significant difference in survival between the 2 groups in our analysis. As shown in Figure 2, the survival curves for patients receiving S-CRT and S-CT showed no divergence, although the S-CRT group had a trend of worse survival. Furthermore, analysis revealed that there were no differences in the local recurrent rates of the S-CRT and S-CT groups, whereas the S-CRT group had higher distant metastasis rates (*P* = 0.038). This suggests that the poor prognosis resulting in DMR may be achieved by adding radiotherapy to postoperative chemotherapy. Several previous analyses suggested that chemotherapy should be given first for a limited stage disease^1,19^ because PSCCE was regarded as a systemic disease with high distant recurrence.^8,21,27^ In our study, a subgroup study was conducted for all 29 limited stage II disease patients who received PORT. There was a tendency for the benefit of DMR between the ER and LR groups, favoring the late radiotherapy group. This may be explained by the systemic chemotherapy given first to control the distant failure for those who received late radiotherapy after surgery, which is similar to the results of the previous analysis.^8,21,27^ Surgical resection frequently leads to a decreased local recurrence rate, whereas local control is usually preserved, and disseminated disease appears rapidly in limited stage patients.^10^ Therefore, patients who received early radiotherapy delayed the adequate cycle of chemotherapy and increased the risk of metastatic recurrence.^12^

Death directly attributable to cancer, such as multiorgan metastasis, is most common in patients with PSCCE. Clinical evidence demonstrated that PSCCE is a systemic disease rather than a local disease due to the high incidence of distant recurrence as well as SCLC.^24,27,30^ In this study, distant metastases occurred most frequently in the liver, lungs, and lymph nodes, and brain metastasis was rare.^12,13^ In the absence of neurology symptoms, brain contrast MRI was not routinely performed. Because of rare brain metastasis, prophylactic cranial irradiation is not routinely recommended according to this analysis.

## CONCLUSION

Our analysis shows that compared with surgery only, adjuvant chemotherapy improves both disease-free survival in PSCCE patients with completely resected LSII disease, whereas adjuvant treatment with postoperative chemotherapy or postoperative chemo-radiotherapy does not improve disease-free survival in completely resected LSI disease. Because potential bias may exist in the present study, larger, prospective randomized clinical trials are warranted to confirm these findings.
